# Oxaliplatin- or irinotecan-based chemotherapy for metastatic colorectal cancer in the elderly

**DOI:** 10.1038/sj.bjc.6601805

**Published:** 2004-04-13

**Authors:** C Alliot, M Barrios

**Affiliations:** 1 Hematology/Oncology Division, General Hospital of Annemasse, BP 525, Annemasse Cedex 74107, France; 2 Laboratory of Biochemistry, Avicenne University Hospital, Bobigny, France

**Sir**,

In the 20 October 2003 issue, Aparicio *et al* (2003) reported about 66 patients with metastasic colorectal cancer aged from 75 to 88 (median, 78) treated with oxaliplatin or irinotecan in combination with either 5-fluorouracil or raltitrexed. The authors state on the feasibility of these regimens in the elderly population. The first point is the limited recruitment by 12 centres during more than 3 years, suggesting highly selected population. It would have been interesting to estimate the proportion of patients receiving no chemotherapy. The main point is the high grade III or IV toxicity rate of 42%. Given the prognosis and cost of these drugs, is this acceptable? Although efficacy is a major goal in some situations including candidates to metastasectomy or parents of young children, chemotherapy remains palliative in nearly all cases. Significant increased toxicity has previously been found in patients aged over 65 years in two phase II study of irinotecan (Rougier *et al*, 1997; Rothenberg *et al*, 1999). Increased toxicity is logically observed in elderly patients given numerous pharmacokinetic changes, including loss of total body water, increased total body fat, or decreased albumin (Wallace and Verbeck, 1983). Oxaliplatin is intensively bound on the erythrocytes (Pendyala and Creaven, 1993). The albumin protein binding for oxaliplatin and SN-38, the active metabolite of irinotecan is greater than 90%. Thus, the volume of distribution may be also increased by anaemia or hypoalbuminaemia. The glomerular filtration rate progressively declines by about 1% each year after the age of 40 years (Evers *et al*, 1995). Thus, dose adaptation of drugs with renal elimination has been proposed in case of creatinine clearance lower than 60 ml min^−1^ (Kintzel and Dorr, 1995). The mean creatinine clearance was about 57 ml min^−1^ in the present study. Polypharmacy also is a major concern. Certain treatments of comorbid illnesses may interfere with chemotherapy, in particular for cytochrome *P*-450 enzyme or conjugation reactions. Irinotecan is extensively metabolised by the cytochrome *P*-450 isoenzymes CYP3A4 and CYP3A5 (Santos *et al*, 2000). For example, CYP3A4 can be induced by carbamazepin or phenytoin and inhibited by macrolides or antifungal imidazoles (Balis, 1986). Numerous drugs such as warfarin, phenytoin or salicylates may displace chemotherapeutic agents from albumin binding sites (Spina and Scordo, 2002). In other words, standard full-dose regimen represents overdosage in a wide fraction of this population. Hepatic impairment due to liver involvement might be a particular concern. Bilirubin might compete with chemotherapeutic agents for albumin binding. Significant elevations in transaminases have been reported under raltitrexed, particularly in case of hepatic metastases or abnormal baseline transaminases levels (Cocconi *et al*, 1998). The liver intervenes by many aspects in the metabolism of irinotecan and SN38, implicating the cytochromes, glucuro-conjugation by UDP-glucuronosyltransferase 1A1 (UGT1A1) and enterohepatic cycling, resulting in wide interpatient variability (Rivory, 2000). High bilirubin and alkaline phosphate levels are associated with toxicity (Raymond *et al*, 2002). Severe toxicity also has been observed in patients with unconjugated hyperbilirubinaemia due to UGT1A1 deficiency (Wasserman *et al*, 1997) encountered in Gilbert's syndrome (5% of the population). Although the present study demonstrates significant efficacy in elderly patients, chemotherapy must be adapted to the diversity of the elderly population. In line with this, certain measures potentially could decrease toxicity such as the adaptation of comedications or correction of anaemia and hypoalbuminaemia. Alkalisation might prevent irinotecan-induced diarrhoea by orientating the intestinal metabolism of SN-38 towards carboxylate form (Ikegami *et al*, 2002).
